# Spontaneous and iatrogenic ovarian hyperstimulation syndrome in the absence of FSHR mutations: a case report of two unexpected cases

**DOI:** 10.1186/s12920-023-01473-3

**Published:** 2023-03-07

**Authors:** Jessica Daolio, Samantha Sperduti, Livio Casarini, Angela Falbo, Caterina Materazzo, Lorenzo Aguzzoli, Maria Teresa Villani

**Affiliations:** 1 Quality and Accreditation Office, Medical Directorate ASMN, Azienda Unità Sanitaria Locale - IRCCS di Reggio Emilia, viale Umberto I 50, 42123 Reggio Emilia, Italy; 2 Unit of Endocrinology, Department of Biomedical, Metabolic and Neural Sciences, University of Modena and Reggio Emilia, via Campi 287, 41125 Modena, Italy; 3 Center for Genomic Research, University of Modena and Reggio Emilia, via Campi 287, 41125 Modena, Italy; 4 Department of Obstetrics & Gynaecology, Azienda Unità Sanitaria Locale - IRCCS di Reggio Emilia, viale Risorgimento 80, 42123 Reggio Emilia, Italy

**Keywords:** Ovarian hyperstimulation syndrome, Cryopreservation, *In vitro* fertilization, Frozen embryo replacement, GnRH agonist, GnRH antagonist

## Abstract

**Background:**

Ovarian hyperstimulation syndrome (OHSS) is a complication of controlled ovarian hyperstimulation (COH). It is a potentially life-threatening condition that usually occurs either after human chorionic gonadotropins (hCG) administration in susceptible patients or as a result of an implanting pregnancy, regardless of whether it was achieved by natural conception or infertility treatments. Despite many years of clinical experience regarding the adoption of preventive measures and the identification of patients at high risk, the pathophysiology of OHSS is poorly understood and no reliable predictive risk factors have been identified.

**Cases presentation:**

We report about two unexpected cases of OHSS following infertility treatments, occurring after freeze-all strategy with embryo cryopreservation approaches. The first case developed spontaneous OHSS (sOHSS), despite efforts to prevent its manifestation by a segmentation approach, including frozen embryo replacement cycle. The second case developed a late form of iatrogenic OHSS (iOHSS), even though the absence of any risk factors. No mutations in the follicle-stimulating hormone (FSH) receptor (FSHR)-encoding gene were detected, suggesting that the high levels of hCG due to the twin implanting pregnancies could be the only triggering factor of OHSS outbreak.

**Conclusion:**

Freeze-all strategy with embryo cryopreservation cannot entirely prevent the development of OHSS, which may occur in its spontaneous form independently from the FSHR genotype. Although OHSS remains a rare event, all infertile patients requiring ovulation induction or controlled ovarian stimulation (COS) may be at potential risk of OHSS, either in the presence or in the absence of risk factors. We suggest closely monitoring cases of pregnancy following infertility treatments in order to provide early diagnosis and adopt the conservative management.

**Supplementary Information:**

The online version contains supplementary material available at 10.1186/s12920-023-01473-3.

## Background

Ovarian hyperstimulation syndrome (OHSS) is a systemic condition characterized by a massive ovarian enlargement and an increased vascular permeability responsible for an abnormal fluid shift from the intravascular space to the extravascular compartments [1, 2]. It is due to an exaggerated response to endogenous and/or exogenous gonadotropins, inducing the secretion of vasoactive mediators by granulosa cells. OHSS displays several features, such as ascites, pericardial effusion, hypovolaemia, oliguria, thromboembolic events and hydroelectrolytic imbalances [3]. The clinical picture defines the severity of the syndrome, which is classified as mild, moderate, severe or critical [4–6].

OHSS is one of the most serious complications related to ovulation induction and controlled ovarian stimulation (COS) protocols during infertility treatments [7]. It usually occurs in women susceptible to human chorionic gonadotropin (hCG), either administrated to trigger oocyte maturation during in vitro fertilization (IVF) cycles or derived from an implanting pregnancy [8, 9]. The prevention of OHSS is preferred over its treatment and relies on the evaluation of risk factors, such as young age, low body mass index, polycystic ovarian syndrome (PCOS), elevated dosages of gonadotropins and history of previous OHSS. Secondary OHSS risk factors are known to be large number of growing follicles on the day of triggering, high number of oocytes retrieved on the day of ovum pick-up, and high levels of serum estradiol on the day of triggering [10]. In patients at risk to develop OHSS, COS protocols with gonadotropins-releasing hormone (GnRH) antagonist followed by GnRH agonist trigger and “freeze-all” strategies are recommended to prevent severe forms of the syndrome [11–13].

To date, OHSS is described based on timing of symptoms presentation. The iatrogenic form (iOHSS) develops after the ovulation triggering mediated by the hCG administration during IVF cycles [14, 15]. Differently, the spontaneous form of OHSS (sOHSS) generally develops between the 8th and 14th week of gestation, independently of whether the pregnancy is achieved by IVF or natural conception [16]. iOHSS includes the early and late forms, that occur 3–7 days and 12–17 days after triggering, respectively; the late form of iOHSS is associated with an implanting pregnancy, in which an excessive amount of pregnancy-derived hCG is produced [14, 15]. Late iOHSS is more likely to be severe than early iOHSS and more difficult to be predicted, based on the ovarian response to COS [15].

hCG molecules play a crucial role in the pathogenesis of both forms of OHSS, as they cross-activate follicle-stimulating hormone (FSH) receptor (FSHR)-dependent signals in granulosa cells, inducing the secretion of vasoactive ovarian mediators, such as the vascular endothelial growth factor (VEGF), as well as other growth factors and cytokines responsible for the development of the syndrome [2, 9, 17–21]. The cross-activation of FSHR due to high levels of hCG is well described in multiple pregnancies and hydatiform moles, as conditions exposing the patient to higher risk of developing sOHSS. Since hCG shares structural similarities with other glycoprotein hormones, such as follicle stimulating hormone (FSH), luteinizing hormone (LH) and thyroid stimulating hormone (TSH), sOHSS may arise as a subsequence of pathological conditions in which these hormones achieve serum concentrations so high to cross-interact with FSHR [22], such as gonadotropin secreting-pituitary adenomas and hypothyroidism.

Genetic investigations conducted in familial and recurrent cases of OHSS demonstrated that some patients could be carriers of *FSHR* gene mutations [23–31], providing a rationale for the molecular mechanism supporting FSHR cross-interaction with other hormones. Mutant FSHRs with increased sensitivity to glycoprotein hormones and enhanced basal activity were described in OHSS patients [27, 32–36]. These mutations fall within the transmembrane helices [27, 33, 35, 37], in the extracellular domain [36, 38] and in the cytoplasmic tail [39, 40] of the receptor, impacting the FSHR density in the cell surface and the activation of intracellular signaling pathways. More recently, a study found that two biallelic heterozygous FSHR mutations were linked to OHSS in a pregnant patient affected by sOHSS, triggered by hCG [22]. The determination of FSHR mutations may be used to classify sOHSS into different types [31, 35], strengthening the relevance of genetic analysis in patients with increased risk of developing the syndrome.

Here we report about two cases of OHSS: a case of sOHSS developed following a frozen embryo replacement cycle and a case of iOHSS that was unexpected, due to the absence of risk factors at COS beginning. The FSHR genotype was determined in both patients.

## Case 1 presentation

A 33-year-old nulligravida woman and her husband presented a 30-months history of primary infertility. Polycystic-like ovaries were identified by transvaginal ultrasound. Her body-mass index (BMI) was 22.22 kg/m^2^. The husband presented a mild oligoastenozoospermia, assessed according to international guidelines [41]. Controlled ovarian hyperstimulation for assisted reproduction was performed using an individualized protocol with gonadotropins in a long GnRH agonist (Enantone® 3.75 mg/ml; Takeda Pharmaceutical Company Ltd., Tokyo, Japan) down-regulated cycle. Ovarian stimulation lasted 10 days: it was achieved by 87.5 IU/die recombinant FSH administrations (Gonal-F®; Merck KGaA, Darmstadt, Germany), accounting for 875 IU total units, which supported the development of 20 growing follicles larger than 14 mm. 5000 IU of highly purified hCG (Gonasi®; IBSA Institut Biochimique SA, Lugano, Switzerland) were used to trigger ovulation and, 36 h later, a total number of 20 oocytes were retrieved during ovum pick-up. Given the high levels of estradiol (4932 pg/ml) on the day of oocyte maturation triggering, the large number of growing follicles and the large number of oocytes retrieved, the risk of OHSS was ascertained. All embryos obtained by intracytoplasmic sperm injection (ICSI) were frozen. A frozen embryo replacement cycle was done four months later, following the recovery of physiological ovarian size. Before embryo transfer, luteal phase support was provided according to a standardized protocol of our Centre: oral administrations of 2 mg estradiol valerate (Progynova®; Bayer AG, Leverkusen, Germany) were given twice daily. Endometrial maturation was monitored by serial ultrasounds beginning from day 12 of patient’s natural period. Daily intravaginal depots of progesterone (Crinone®; Merck KGaA) were initiated when endometrial thickness was 8–12 mm. Embryo transfer was performed 3 days after the first administration of progesterone. Medical treatment was continued until serum hCGβ dosing, that was scheduled 14 days after the embryo transfer. Two, 48-h consecutive and positive hCGβ tests were performed to confirm implanting pregnancy. The transvaginal ultrasound was performed 4 weeks later, revealing the presence of two dichorionic diamniotic gestational sacs.

The patient was hospitalized at 12th week of gestation for pelvic pain and occasional respiratory distress. Her familial, medical and gynecological history was unremarkable apart from the infertility treatment. Clinical examination revealed no abdominal distension or acute abdomen. Ultrasonography showed the ongoing dichorionic-diamniotic gestation and bilateral enlarged ovaries; the right ovary measured 11.78 × 6.39 cm, while the left one 9.73 × 8.14 cm. No free fluids were identified in the pelvis and abdomen, apart from a light fluid spilling in the right paracolic gutter. Echocardiogram was normal. Clinical blood parameters were: 1096 per ml white blood cell count, 10.0 g/dl hemoglobin, 31.2% hematocrit, 256,000 platelets per ml, 3.68 g/dl albumin and 125 of 79.3 IU/ml albumin-corrected calcium.

A diagnosis of mild sOHSS was formulated and the patient was supportively managed with 4000 IU/die low molecular weight heparin (Clexane®; Sanofi, Paris, France), as a thromboprophylaxis therapy for the hydrolytic balance combined with a salt-restricted diet. During the hospitalization, vital signs were evaluated every 12 h. A complete physical evaluation was conducted daily in order to record and monitor patient’s weight, abdominal circumference, fluids intake and output, electrolytes concentrations, complete blood count, liver enzymes dosages and urinary parameters. Overall, the course of the patient’s stay was regular with improved biochemical parameters, abdominal circumference and body weight. The patient was discharged on the fifth day after that the disease did not evolve to a more severe stage. During follow-up, sonography showed regular progression of pregnancy, except for the presence of obstetric cholestasis. A cesarean section was performed at 36 gestational weeks with the live birth of a healthy male and a healthy female weighing 3080 g and 2189 g, respectively. The patient had no post-partum complications.

DNA sequencing of the *FSHR* gene was performed by using genomic DNA extracted from patient’s peripheral lymphocytes as described below (see the “DNA sequencing” section). No FSHR mutations were identified.

## Case 2 presentation

A 37-year-old nulligravida female and her husband presented a one-year and a half history of idiopathic infertility. Her BMI was 19.10 kg/m^2^. The husband presented astenoteratozoospermia, assessed according to international guidelines [41]. Controlled ovarian hyperstimulation was performed using an individualized protocol with gonadotropins, in a GnRH agonist (Decapeptyl® 3.75 mg; Ipsen; Italy) down-regulated cycle. Ovarian stimulation lasted 16 days by 100 IU/die recombinant FSH administrations (Gonal-F®, Merck KGaA), accounting for 1300 IU total units, and it supported the development of 6 growing follicles larger than 14 mm. 10,000 IU highly purified hCG (Gonasi®, IBSA Institut Biochimique SA) were used to trigger ovulation and, 36 h later, 5 oocytes were retrieved. On the day of triggering, the concentration of serum estradiol was 2259 pg/ml. Two viable embryos were obtained by ICSI and transferred at the day 3 of cleavage stage. The luteal phase was supported by daily intravaginal depots of progesterone (Crinone®; Merck KGaA) until serum hCGβ determination, which was scheduled 14 days after the embryo transfer.

A week after the embryo transfer, corresponding to 13th days after the hCG-induced triggering, the patient was hospitalized due to pelvic and abdominal pain, and swelling. Her medical history was unremarkable, apart from the use of oral contraceptives for several years and an endometrial polypectomy carried out ten years before the infertility treatment. Clinical examination revealed the presence of Blumberg’s sign. Ultrasonography revealed regular uterine morphology, the presence of fluids in the pelvis, Douglas tract and both paracolic gutters. The right ovary measured 8.7 × 7.9 cm and the left one 7.9 × 5.8 cm. Echocardiogram was normal. Clinical blood parameters were: 1273 per ml white blood cell count, 17.9 g/dl hemoglobin, 51.9% hematocrit, 229,000 platelets per ml and 3.39 g/dl albumin.

The suspicion of hemoperitoneum was initially considered prior to finally formulating the diagnosis of late iOHSS. Accordingly, the patient was managed with 4000 IU/die low molecular weight heparin as a thromboprophylaxis (Clexane®; Sanofi), 40 mg/die albumin and a therapy for the hydrolytic balance. On second day, the implanting pregnancy was revealed by hCGβ determination and confirmed by a second, positive hCGβ test 48 h later. During the hospitalization, vital signs were evaluated every 12 h. A complete physical evaluation was conducted daily in order to record and monitor patient’s weight, abdominal circumference, fluids intake and output, electrolytes concentrations, complete blood count, liver enzymes dosages and urinary parameters. Overall, the course of patient’s stay was regular with improved biochemical parameters, abdominal circumference and body weight. The patient was discharged on the seventh day after the disease did not progress to a more severe stage.

Dichorionic diamniotic pregnancy was confirmed by transvaginal ultrasound 4 weeks after the second hCGβ assay. The gestational period progressed regularly during follow-up, until cesarean section delivery, that was scheduled at the 35th week of gestation. Two healthy males weighing 2464 and 2836 g were born. The patient had no post-partum complications.

No FSHR mutations were identified by DNA sequencing.

### DNA sequencing

Patients’ *FSHR* gene was sequenced using the Sanger’s method, as previously described (Lazzaretti et al., 2019). Genomic DNA was extracted from blood samples using the automated extractor EZ1 Advanced XL (Qiagen, Hilden, Germany) and quantitatively determined by a NanoDrop™ 2000 spectrophotometer (Thermo Fisher Scientific, Waltham, MA, USA). *FSHR* gene-encoding regions were amplified using the Expand High Fidelity PCR System (Roche), except for a 5’ segment of exon 10, which was amplified using the AmpliTaq™ 360 DNA Polymerase (Thermo Fisher Scientific), according to the manufacturer’s protocols. Specific primer sequences were designed using the *FSHR* NCBI Reference Sequence as a template (NG_008146.1) and obtained by *de novo* synthesis (Thermo Fisher Scientific). Amplicon length and the specific annealing temperature are reported (Table 1).

**Table 1:** Primer sequences used for FSHR DNA Sanger’s sequencing

position	forward primer	reverse primer	amplicon lenght	annealing temperature
Exon 1	5’-CATCCCTTGGTGGGTCACATG-3’	5’-AAATGCCAGCCATGCAGTTG-3’	336 bp	59 °C
Exon 2	5’-AGACAGGATGAAAAGAGAGAATG-3’	5’-TTGAGGCATTCACTCACAGC-3’	263 bp	57 °C
Exon 3	5’-GCCACAGCCTTCGACTTATTC-3’	5’-GCCTCCCAGGAATGTAGAAG-3’	367 bp	59 °C
Exon 4	5’-GCACAGCTTAGTGTGATAAAAGGC-3’	5’-GTGGGGGTACCAAACTACATG-3’	302 bp	59 °C
Exon 5	5’-CTCTGAGGAATCAACAGCTTTTAAG-3’	5’-GGGCAAGACAGATACTGAG-3’	334 bp	53 °C
Exon 6	5’-GTCTGCAATTCCATTTGTAAGAAC-3’	5’-ATCAAATGTTACTCTGTTGG-3’	327 bp	50 °C
Exon 7–8	5’-TACAGCAATAAATCAGTCTTCCTCC-3’	5’-GAGAGTTGACTTCTAACTTACAC-3’	489 bp	59 °C
Exon 9	5’-GAAGGACCAGGACTCCTACAGAAC-3’	5’-TGCCTGAGCAGGGCTTAAAG-3’	415 bp	57 °C
Exon 10, 5’ segment	5’-GCTATACTGGATCTGAGATG-3’	5’-ATCCAGCCCATCACCATGAC-3’	680 bp	62 °C
Exon 10, 3’ segment	5’-AGCTGGACTGCAAGGTGCAG-3’	5’- TGTAGAAGCACTGTCAGCTC-3’	752 bp	59 °C

Abbreviations: bp, base pair.

PCR products were purified using the High Pure PCR Product Purification Kit (Merck KGaA, Darmstadt, Germany). 20 ng of purified PCR product were sequenced by the BigDye Terminator v3.1 Cycle Sequencing Kit (Life Technologies, Carlsbad, CA, USA), using 3.5 pmol/reaction of the primer sequence solution, in a total reaction volume of 20 µl. Reactions were run according to the following thermal cycler conditions: first denaturation stage at 96 °C for 30 s; 30 cycles of denaturation at 96 °C for 10 s, annealing at 50 °C for 5 s and extension at 60 °C for 4 min. After cycle sequencing, reactions were purified with sodium acetate/ethanol precipitation and separated by capillary electrophoresis on the automatic sequencer ABI 3130 Genetic Analyzer (Applied Biosystems, Paisley, United Kingdom). Data were processed using the Sequencing Analysis Software (Applied Biosystems, Paisley, UK) and compared to those from the online *FSHR* template sequence (NG_008146.1).

**Table 2:** Summary of published OHSS cases related to different clinical contests

Reference	Genotype	Phenotype	Context details	Patient’s condition		Onset of symptoms	Outcome
*Multiple pregnancy*							
Gil Navarro N, 2017	No mutation		Ovarian torsion	Natural twin pregnancy		11th	
Agrawal NR et al., 2012	Not tested			Natural triplet pregnancy		6th	Abortion induction
Sugaya S and Hiroi T, 2012			Previous ovulation induction plus FSH	Natural quadruplet pregnancy		3rd	Abortion induction
*Molar pregnancy*							
Cohen E et al., 2019	Not tested			Hydatiform mole		12th	
Tsubokura H et al., 2019	Not tested			Hydatiform mole		9th	
Alhalabi K et al., 2016	Not tested			Hydatiform mole		14th	
Gaggero Cr et al., 2016	Not tested			Hydatiform mole		12th	
Wu X et al., 2015	p.Ala307Thr p.Ser680Asn	Hypersensitivity to hCG and TSH; constitutive activity		Hydatiform mole		9th	
Teo UL et al., 2013	Not tested			Hydatiform mole		12th	
Zhou X and Duan Z, 2012	Not tested			Hydatiform mole		16th	
Rachad M et al., 2011	Not tested			Hydatiform mole		12th	
Strafford M et al., 2009	Not tested			Hydatiform mole		12th	
Arora R et al., 2008	Not tested			Hydatiform mole			
Ludwig M et al., 1998	Not tested			Hydatiform mole		15th	
*Pituitary adenoma*							
Broughton C et al., 2018	Not tested			Pituitary adenoma			
Oueslati I et al., 2016	Not tested			Pituitary adenoma			
Halupczok J et al., 2014	Not tested			Pituitary adenoma			
Kawaguchi T et al., 2013	Not tested			Pituitary adenoma			
Kanaya M et al., 2012	Not tested			Pituitary adenoma			
Garmes HM et al., 2012	Not tested			Pituitary adenoma			
Macchia E et al., 2012	Not tested			Pituitary adenoma			
Gryngarten MG et al., 2010	Not tested			Pituitary adenoma		Post-menarcheal girl	
Baba T et al., 2009	No mutation			Pituitary adenoma		After positive pregnancy test	
Castelo-Branco C et al., 2009	Not tested			Pituitary adenoma			
Cooper O et al., 2008	Not tested			Pituitary adenoma			
Ghayuri M and Liu JH, 2007				Pituitary adenoma			
Knoepfelmacher M et al., 2006	Not tested			Pituitary adenoma			
Kihara M et al., 2006	Not tested			Pituitary adenoma			
Roberts JE et al., 2005	Not tested			Pituitary adenoma			
Maruyama T et al., 2005	p.Met512Ile (Uchida S et al., 2013)	Inactive mutant		Pituitary adenoma			
Murakami T et al., 2004	Not tested			Pituitary adenoma			
Murata Y et al., 2003	Not tested			Pituitary adenoma			
Castelbaum AJ et al., 2002	Not tested			Pituitary adenoma			
Shimon I et al., 2001	Not tested			Pituitary adenoma			
Pentz-Vidovíc I et al., 2000	Not tested			Pituitary adenoma			
Vӓlimӓki MJ et al., 1999	Not tested			Pituitary adenoma			
Christin-Maitre S et al., 1998	Not tested			Pituitary adenoma			
Djerassi A et al., 1995	Not tested			Pituitary adenoma			
*Hypothyroidism*							
Kim SJ et al., 2017	Not tested			Hypothyroidism			
Ilanchezhian S et al., 2015	Not tested			Hypothyroidism			
Rajaram S et al., 2015	Not tested			Hypothyroidism			
Lodh M et al., 2014	Not tested			Hypothyroidism		Admission 15 days after ET	iOHSS
Erol O et al., 2013	No mutation			Hypothyroidism			
Langroudi RM et al., 2013	Not tested			Hypothyroidism			
Kanza RE et al., 2013	Not tested		PCOS Pituitary hyperplasia	Hypothyroidism			
Sridev S and Barathan S, 2013	Not tested			Hypothyroidism		9th	
Hedayati Emami MH et al., 2012	Not tested		Familial	Hypothyroidism			
Akbay E et al., 2010	Not tested		Recurrent sOHSS during pregnancies	Hypothyroidism		10th and 12th	
Edwards-Silva RN et al., 2008	Not tested			Hypothyroidism		10th	
Borna S and Nasery A, 2007	Not tested		sOHSS	Hypothyroidism		20th	
Sultan A et al., 2006	No mutation			Hypothyroidism			
Guvenal F et al., 2006	Not tested			Hypothyroidism			
Mousavi AS et al., 2005	Not tested			Hypothyroidism			
Taher BM et al., 2004	No mutation (De Leener A et al., 2006)	High levels of TSH		Hypothyroidism			
Corsado CG et al., 1999				Hypothyroidism		12th	
Nappi RG et al., 1998	No mutation (De Leener A et al., 2006)	High levels of TSH		Hypothyroidism		12th	
Chen CP et al., 1996	Not tested			Hypothyroidism			
Rotmensch S and Scommegna A, 1989	Not tested			Hypothyroidism			
*Natural conception*							
Nakatsuka M et al., 2019	Not tested			Natural conception	After delivery		
Morotti E and Battaglia C, 2019	Not tested			Natural conception	8th		
Rastin Z et al., 2019	Not tested			Natural conception	8th		
Lazzaretti C et al., 2019	p.Asn106His p.Ser128Tyr	Inactive mutant Hypersensitivity to hCG		Natural conception	11th		
Celik S et al., 2019	Not tested			Natural conception	9th - continuation of disease after abortion		
Topdagi Yilmaz EP et al., 2018	p.Ser128Tyr p.Ala307Thr p.Ser680Asp	Hypersensitivity to hCG Hypersensitivity to hCG and TSH; constitutive activity		Natural conception	12th		
Topdagi Yilmaz EP et al., 2018	p.Ser128Tyr	Hypersensitivity to hCG		Natural conception		10th	
Cabar FR, 2016	Not tested			Natural conception		12th	
Osaikhuwuomwan JA and Osemwenkha AP, 2016	Not tested			Natural conception		10th	
Davoudian P, 2015	Not tested			Placental dysplasia		16th	Miscarriage
Chauhan AR et al., 2015	p.Thr449Asn	Not tested		Natural conception		8th	
Munshi S, 2014	Not tested		Ovarian torsion	Natural conception		9th	
Di Carlo C, 2013 (patient’s cousin in Di Carlo C et al., 2012)	p.Thr449Ala (Montanelli L et al., 2004 J Clin Endocrinol Metabol)	Hypersensitivity to hCG and TSH	Recurrent and familial	Natural conception		8th (both patient and cousin)	Patient with ovarian torsion two years after delivery (Di Carlo C et al., 2015)
Panagiotopoulou N, 2013	p.Ile545Thr	Hypersensitivity to hCG and TSH; constitutive activity	Recurrent and familial	Natural conception		10th	
Di Carlo C, 2012	p.Thr449Ala (Montanelli L et al., 2004 J Clin Endocrinol Metabol)	Hypersensitivity to hCG and TSH	Recurrent and familial	Natural conception in a patient and her cousin		7th (patient) – not reported (cousin)	Both abortion induction
Kanagalingam MG, 2011	Not tested			Natural conception		10th	
Irvine LM, 2011	Not tested			Natural conception		8th	
Ahmed Kamel RM, 2010	Not tested			Natural conception		12th	
Dieterich M et al., 2010	p.Asp567Asn	Increased basal activity		Natural conception		12th and 10th	
Lussiana C et al., 2009	p.Thr307Thr p.Asn680Asn	polymorphism		Natural conception		Abortion at 22th	
Dasanayake DL et al., 2009	Not tested		Recurrent and familial	Natural conception		9th	
O’Brien K et al., 2009	Not tested			Natural conception		17th	Abortion induction
Lovgren TR et al., 2009	Not tested		recurrent	Natural conception		8th	
Michaelson-Cohen R et al., 2008	p. Ala307Thr p.Ser680Asn	Hypersensitivity to hCG and TSH; constitutive activity		Natural conception		10th	
He C et al., 2008	Not tested			After delivery			
Oztekin O et al., 2006	Not tested	High levels of hCG		Natural conception		9th	
Cepni I et al., 2006	p.Ser128Tyr (De Leener A et al., 2008 HUM MUTAT)	Hypersensitivity to hCG		Natural conception		11th	
Eftekhar Z et al., 2005	Not tested		Ovarian torsion	Natural conception		11th	
Haimov-Kochman R et al., 2004	No mutation (De Leener A et al., 2006)	High levels of hCG		Natural conception		13th	
Baksu A et al., 2004	Not tested		Ovarian torsion	Natural conception		10th	
Suzuki S, 2004	p.Asp567Gly (Montanelli L et al., 2004 Mol Endocrinol)	Hypersensitivity to hCG and TSH; constitutive activity		Natural conception		11th	
	No mutation (De Leener A et al., 2006)	High levels of hCG		Natural conception		6th	
Vasseur C et al., 2003	p.Thr449Ile	Hypersensitivity to hCG	Recurrent and familial	Natural conception		8th	
Chae HD et al., 2001	p.Ile545Thr (De Leener A et al., 2006)	Hypersensitivity to hCG and TSH; constitutive activity		Natural conception		12th	
Akerman FM et al., 2000		hCG/LH receptor investigated		Natural conception		12th	
Todros T et al., 1999	Not tested		Factor V Leiden mutation	Natural conception		10th	
Di Carlo C et al., 1997	p.Thr449Ala (Montanelli L et al., 2004 J Clin Endocrinol Metabol)	Hypersensitivity to hCG and TSH	Recurrent, familial	Natural conception		10th	
Edi-Osagie EC and Hopkins RE, 1997	p.Ile545Thr (mother of patient’s from Panagiotopoulou N et al., 2013)	Hypersensitivity to hCG and TSH; constitutive activity	Recurrent and familial	Natural conception			
Abu-Louz SK et al., 1997	Not tested			Natural conception		12th	
Ayhan A et al., 1996	Not tested			Natural conception		12th	
Lipitz S et al., 1996	Not tested			Natural conception		10th	
Olatunbosun OA et al., 1996	p.Asp567Asn (Smits G et al., 2003)	Increased basal activity	Recurrent in a PCOS woman	Natural conception			sOHSS
Zalel Y et al., 1995			Recurrent in a PCOS woman	Natural conception			sOHSS
Zalel Y et al., 1992			PCOS	Natural conception			sOHSS
Rosen GF and Lew MW, 1991				Natural conception			sOHSS
*Non-pregnant women and virgin girls*							
Hugon-Rodin J et al., 2017	p.Arg634His	Inactive mutant	Recurrent	Non pregnant			
Attia L et al., 2007			Bilateral ovarian masses	Not pregnant			
Sahin L and Yavuzcan A, 2013	Not tested		Recurrent	Virgin girl			
*IVF*							
Castillo J et al., 2019	Not tested			Egg-donation IVF cycle with concomitant pregnancy		Admission 4 days after ovum pick-up (5th week of gestation)	iOHSS
Cohen E et al., 2019	Not tested			COS		Admission in the evening of ovum pick-up	iOHSS
Orvieto S et al., 2017	Not tested		Extrauterine pregnancy	COS		Admission 6 days after ovum pick-up	iOHSS
Pereira N et al., 2017	Not tested		PCOS	COS		Admission 2 days after ovum pick-up	iOHSS
Kim MK et al., 2014	p.Ala307Thr p.Ser680Asn	Hypersensitivity to hCG and TSH; constitutive activity	PCOS	Twin pregnancy following a FER cycle		11th	sOHSS - Preterm delivery and Persisted 6 weeks after delivery
Kim J et al., 2012	Not tested			IVF cycle (fertility preservation)		Admission 5 days after hCG trigger	iOHSS
Taniguchi LU et al., 2011	Not tested		Previous OHSS after IVF	IVF (fresh cycle) twin pregnancy		5th	Very late iOHSS
Crochet JR et al., 2011	Not tested			IVF cycle		Admission 10 days after hCG trigger	iOHSS
Zech NH et al., 2005	Not tested			IVF (fresh cycle) pregnancy		6th	Very late iOHSS

Abbreviations: COS, controlled ovarian stimulation; FER, frozen embryo replacement; hCG, human chorionic gonadotropins; IVF, in vitro fertilization; iOHSS, iatrogenic ovarian hyperstimulation syndrome; LH, luteinizing hormone; OHSS, ovarian hyperstimulation syndrome; PCOS, polycystic ovary syndrome; sOHSS, spontaneous ovarian hyperstimulation syndrome; TSH, thyroid stimulating hormone.

## Discussion

We reported two cases of OHSS with apparent no main risk factors and *FSHR* gene mutations, suggesting the use of a conservative clinical approach in which all women undergoing COS should be considered at potential risk of developing the syndrome.

In the first patient described in the present report, two primary risk factors were suggestive of OHSS development: the young age and the presence of polycystic-like ovaries. On the day of ovum pick-up, the presence of further, secondary risk factors implied the application of the freeze-all policy to prevent OHSS. The patient’s compliance to the medical treatment during hospitalization allowed the physiological resolution of the syndrome without progression of OHSS to a more severe stage. Interestingly, no *FSHR* gene mutations were found, prompting us to hypothesize the presence of other, unknown risk factors. To our best knowledge, only another patient undergoing IVF and frozen embryo replacement cycle has been reported so far, and in which sOHSS occurred. This patient differed from ours as the DNA sequencing revealed two *FSHR* activating mutations potentially causative of the syndrome [42]. However, in both these patients, the embryo freeze-all strategy did not prevent the development of OHSS, highlighting the relevance of a close monitoring of pregnancies achieved by a frozen embryo replacement cycle, in the presence of risk factors at the beginning of COS.

The second case presented in this report unexpectedly developed a late form of iOHSS, in the absence of any primary or secondary risk factor, apart from a low BMI. The dose of gonadotropin administered was set considering the patient’s BMI and ovarian features. In addition, at the time of hospitalization, the ovaries were within the physiological range. We excluded a genetic predisposition to high OHSS, as no *FSHR* gene mutations were found. We may assume that our two cases strengthen the relevance of a careful evaluation of patients’ features before COS to identify potential OHSS risk factors. Since the prediction of OHSS occurrence, as well as of its degree of severity is practically challenging, all women undergoing COS should be considered at risk of developing the syndrome.

Pregnancies complicated by OHSS may be at increased risk of pre-eclampsia and preterm delivery [43, 44]. Furthermore, pregnancies achieved by IVF in which severe form of OHSS has been developed could have an increased risk of preterm birth [45]. Despite both pregnancies were achieved by IVF treatments, the two patients described in the present report experienced only a mild form of the syndrome and faced a preterm delivery due to the twin gestation. Since no *FSHR* gene mutations were found, we may hypothesize that the high levels of hCG linked to twin pregnancies would be the main responsible for OHSS. Our conclusion is in contrast with that from a previous study stating that elevated hCG cannot induce sOHSS as a single factor [46]. Moreover, genetic screenings and in vitro characterizations of the *FSHR* gene were suggested to achieve an optimized, pharmacogenetic approach to assisted reproduction [22, 47]. We can not exclude unknown factors additionally involved in the syndrome pathogenesis in our two patients. In this context, we may suppose that polymorphisms within other genes regulating the ovarian response to gonadotropins, such as those encoding for kinases, growth factor receptors and intracellular interactors of FSHR [48], may be of potential interest to evaluate OHSS risk, especially in familial or recurrent cases. In fact, the cases described in this report would support the concept of a genetic predisposition for OHSS [29, 30].

OHSS is one of the most serious complications affecting women undergoing ovulation induction or COS for infertility treatments. However, it may affect also non-pregnant women, virgin girls as well as women affected by diseases, such as gonadotropins-secreting adenoma, molar pregnancies, or hypothyroidism (Table 2). All these clinical contests were collected in the present report to provide, to our best knowledge, the widest summary of cases published so far (Table 2).

Despite the severe form of OHSS occurs in 0.2–1.2% of the IVF cycles, the true incidence of the syndrome remains unknown because the reporting of mild or moderate cases is not mandatory [49]. In order to assist in reporting new cases and make easier comparisons of data from different studies, two classification systems were introduced: the first one regards a pathophysiological classification of OHSS based on the presence or absence of *FSHR* mutations [31], whereas the second one concerns the classification of OHSS severity based on clinical and laboratory features [5, 6].

The prevention of OHSS is preferred over its treatment and every attempt should be made in order to identify patients at high risk. Several primary and secondary risk factors for OHSS have been identified, but the sensitivity and specificity for predicting OHSS is variable [49, 50]. Therefore, some degree of ovarian hyperstimulation may be considered as usual during the clinical procedure and it is difficult to discriminate between symptoms of mild hyperstimulation and the disease. According to the European Society of Human Reproduction and Embryology (ESHRE), the segmentation approach, including GnRH agonist trigger in a GnRH antagonist cycle, embryo cryopreservation and frozen embryo replacement cycles, should be adopted in patients at high risk for OHSS [51]. However, OHSS may occur although efforts to identify risk factors and prevent the syndrome are made, challenging the application of preventive measures [13, 42, 52, 53].

Several studies described the management of OHSS in pregnancy [54, 55]. In this case, the treatment of OHSS is conservative and should be defined according to the severity of clinical signs, blood clinical parameters and radiological examinations [56]. The main medical and surgical treatment were extensively described previously [54, 55, 57–60]. Recently, it has been also proposed that the therapeutic principles for OHSS should be consistent with those of the intra-abdominal hypertension syndrome therapy, but further research is needed in this field [61]. In our two cases, the severity of OHSS was mild and moderate, and did not cause any pregnancy complications. This endorses the concept that a well-timed and prompt diagnosis of OHSS helps supporting the patient’s management, especially in case of OHSS recurrence during further pregnancies [23, 24].

## Conclusions

Although OHSS remains a rare event, the present report demonstrates that this syndrome could develop atypically in the absence of recognizable risk factors, despite the adoption of preventive measures during the clinical treatment. Since all women undergoing COS should be considered at potential risk of developing the syndrome, we suggest the use of careful screenings for a conservative clinical approach.

## Electronic supplementary material

Below is the link to the electronic supplementary material.

Supplementary Material 1

## Data Availability

Data and materials are available upon reasonable request to the corresponding author.

## Abbreviations

OHSS: Ovarian hyperstimulation syndrome
COH: Controlled ovarian hyperstimulation
hCG: Human chorionic gonadotropins
sOHSS: Spontaneous ovarian hyperstimulation syndrome
iOHSS: Iatrogenic ovarian hyperstimulation syndrome
FSH: Follicle-stimulating hormone
FSHR: Follicle-stimulating hormone receptor
IVF: in vitro fertilization
PCOS: Polycystic ovary syndrome
GnRH: Gonadotropins-releasing hormone
VEGF: Vascular endothelial growth factor
LH: Luteinizing hormone
TSH: Thyroid-stimulating hormone
BMI: Body-mass index
ICSI: Intracytoplasmic sperm injection
