# Supplementary material for: Spontaneous and iatrogenic ovarian hyperstimulation syndrome in the absence of FSHR mutations: a case report of two unexpected cases
Source: BMC Med Genomics. 2023 Mar 7;16:45. doi: 10.1186/s12920-023-01473-3 (PMC9990314; doi:10.1186/s12920-023-01473-3)
Supplement: Supplementary file 1 — Supplementary Material 1 [file 12920_2023_1473_MOESM1_ESM.pdf]

# CARE Checklist (2013) of information to include when writing a case report

(cc) BY-NC-ND

| Topic | Item | Checklist item description | Reported on Page |
|---|---|---|---|
| Title | 1 | The words "case report" should be in the title along with the area of focus | 1 |
| Key Words | 2 | 2 to 5 key words that identify areas covered in this case report | 2 |
| Abstract | 3a | Introduction—What is unique about this case? What does it add to the medical literature? | 28-42 |
| | 3b | The main symptoms of the patient and the important clinical findings | 28-42 |
| | 3c | The main diagnoses, therapeutics interventions, and outcomes | 28-42 |
| | 3d | Conclusion—What are the main "take-away" lessons from this case? | 43-48 |
| Introduction | 4 | One or two paragraphs summarizing why this case is unique with references | 54-107 |
| Patient Information | 5a | De-identified demographic information and other patient specific information | 109-197 |
| | 5b | Main concerns and symptoms of the patient | 109-197 |
| | 5c | Medical, family, and psychosocial history including relevant genetic information (also see timeline) | 109-197 |
| | 5d | Relevant past interventions and their outcomes | 109-197 |
| Clinical Findings | 6 | Describe the relevant physical examination (PE) and other significant clinical findings | 109-197 |
| Timeline | 7 | Important information from the patient's history organized as a timeline | 109-197 |
| Diagnostic Assessment | 8a | Diagnostic methods (such as PE, laboratory testing, imaging, surveys) | 199-224 |
| | 8b | Diagnostic challenges (such as access, financial, or cultural) | Na |
| | 8c | Diagnostic reasoning including other diagnoses considered | 109-197 |
| | 8d | Prognostic characteristics (such as staging in oncology) where applicable | Na |
| Therapeutic Intervention | 9a | Types of intervention (such as pharmacologic, surgical, preventive, self-care) | 109-197 |
| | 9b | Administration of intervention (such as dosage, strength, duration) | 109-197 |
| | 9c | Changes in intervention (with rationale) | 109-197 |
| Follow-up and Outcomes | 10a | Clinician and patient-assessed outcomes (when appropriate) | 109-197 |
| | 10b | Important follow-up diagnostic and other test results | 109-197 |
| | 10c | Intervention adherence and tolerability (How was this assessed?) | Na |
| | 10d | Adverse and unanticipated events | Na |
| Discussion | 11a | Discussion of the strengths and limitations in your approach to this case | 289-298 |
| | 11b | Discussion of the relevant medical literature | 266-288 |
| | 11c | The rationale for conclusions (including assessment of possible causes) | 301-305 |
| | 11d | The primary "take-away" lessons of this case report | 301-305 |
| Patient Perspective | 12 | When appropriate the patient should share their perspective on the treatments they received | Na |
| Informed Consent | 13 | Did the patient give informed consent? Please provide if requested | Yes X No ☐ |
